# Experimental testing of irradiated engine oil using an e-beam

**DOI:** 10.1038/s41598-023-36693-1

**Published:** 2023-06-12

**Authors:** Martin Zachar, Jan Furch

**Affiliations:** 1 University of Defence, Faculty of Military Technology, 662 10 Brno, Czech Republic; 2 Progresa Final SK, Ltd., 811 08 Bratislava, Slovak Republic

**Keywords:** Mechanical engineering, Experimental nuclear physics

## Abstract

Engine oil forms a complex system with a number of features. These features consist of hydrocarbons, various natural, or synthetic polymers. The irradiation of polymers becomes an integral part of modern industry. The lubrication, charge, thermal and cleaning requirements, which engine oils are expected to meet, are often chemically contradictory and manufacturers have to decide to compromise. Electron accelerators are widely used to enhance polymer properties. By applying radiation technology, it is possible to increase the desirable properties of polymers while leaving other properties at their original value. The paper focuses on combustion engine oil modified by e-beam. The assessed engine oil has a hydrocarbon base, which, from the chemical point of view, is processed as a polymer during the irradiation process. In this paper we have compared selected properties of the conventional and the irradiated engine oil during two exchange intervals. We have examined appropriate dose, dose rate, irradiation volume and a container on a single accelerated electrons energy. The examined oil properties were of a physical and physico-chemical nature, and included mainly kinematic viscosity, viscosity index, the total base number, soot content, oxidation, sulfation, significant chemical elements and wear particles. Every oil attribute is compared with its original value. The main objective of this paper is to demonstrate that the application of e-beam is an adequate method used to enhance engine oil properties to achieve cleaner engine running, and extend engine oil lifetime.

## Introduction

Particle accelerators represent modern technologies used for various applications in science and industry. Electron accelerators belong to the most used in industry. Accelerated electron applications have recently recorded a dynamic increase in polymer production and development. The mentioned technology, however, has not been commonly used in the oil industry despite its significant potential.

Nowadays, combustion engines continually push the boundaries of technology and engineering. They are smaller and more efficient, characterized by the lowest possible performance loss.. Advanced combustion engines challenge the oil with increasing pressure. These intense pressures cause greater friction, which can lead to the loss of up to 10% of the performance of the combustion engine^1^. The most of the engine wear occurs while the engine warms up. The combustion engine oil properties of the utmost importance include friction coefficient, chemical stability, and optimal kinematic viscosity as the engine starts, so it could flow in a shorter time through critical engine parts. The second most important factor is a higher viscosity index, so the engine oil kinematic viscosity should be more independent of temperature. There are many significant properties of engine oil because engine oil is undoubtedly the most technologically complex oil product whose properties are given by a number of often conflicting technical requirements. All kinds of combustion engine oils have inhibitors and additives for various reasons. Most of these oil enhancements are methods of chemical way to raise the engine oil classification. The aim of this paper is to focus on the possibility of physical modification in addition to subsequent chemical additivation. The irradiation of oils is also dealt with in the article^2^, where the effect of irradiation on the change in oil quality has been manifested. The authors achieved the results which demonstrate that oil irradiation led to oxygen incorporation, the formation of oxygenated hydrocarbons, and higher oil viscosities. Oil irradiation was associated with decreased dispersant efficacy, of the efficiency falling from 80 to < 50% in the baffled flask test after more than 3 days of irradiation.

The increase in photo-oxidation-induced viscosity seems to lead to the decrease in dispersant effectiveness. The detailed analysis of the measured data demonstrates that substantial differences are detectable in the spectra of fresh and used oil. Our results manifest that the Zn–S bonds of the ZDDP are decomposed during the operation, resulting in Zn–O bonds instead. Furthermore, six-fold Fe–O bonds similar to those of Fe_2_O_3_ are found within the used oil, suggesting the presence of debris from the anti-wear film of lubricated motor parts in the used oil^3^.

The introduced publication^4^ includes an attempt to link different techniques such as laser and X-ray fluorescence (XRF). Engine oil samples were irradiated with laser and X-rays, which were later diagnosed and analyzed. The X-ray fluorescence technique was used to analyze and determine the concentration of various nanoparticles in engine oil. The paper focuses on investigating data for e-beam radiation applied to combustion engine oil. It is necessary to know the basics of electron radiation in relation to the objectives of the experiment. The presented work addresses a relatively unexplored topic—the theoretical part I takes into account the irradiation of polymers as a whole which behave in the same way as liquid hydrocarbons in the discussed issue. Radiation treatment of polymer properties is a common method in technical practice. Despite the information mentioned above, the irradiation of engine oils is an unexplored area. There are certain published works, however, which introduce the explored issue^5–7^.

## Methodology of research

It is necessary to create a priority list of properties that we will focus on during the gradual research and modification of lubricating media. In relation to the theoretical assumptions and technical possibilities of the current production of engine oils for internal combustion engines, we determined a sequence of priorities focused on the viscosity index, taking into account the type and the number of particles released into the lubricating medium during engine operation and the alkaline reserve during mileage in the experimental measurement in operation. While setting the radiation modification of the lubricating medium, the stated properties of the engine oil will have a higher priority than other monitored quantities. Ptasinsky's results^8, 9^ and other theoretical knowledge^10–12^ are a great prerequisite for achieving the highest efficiency of treatment of the given engine oil with electron radiation in the 10–60 kGy range.

The presented study is carried out with the commercially available Castrol EDGE engine oil, having the SAE 5W-40 specification, ACEA C3, API SN/CF, VW 505.01. The replacement interval of the mentioned engine oil according to the manufacturer's recommendations is 15,000 km. The data on the mentioned engine oil are shown in Table 1.

**Table 1:** Characteristics of engine oil Castrol EDGE 5W40 C3^1^.

Attribute	Method	Unit	Value
Density @ 15 °C, relative	ASTM D4052	g/ml	0.851
Kinematic viscosity, 100 °C	ASTM D445	mm^2^/s	13.2
Kinematic viscosity, 40 °C	ASTM D445	mm^2^/s	77
Viscosity index	ASTM D2270	–	176
Pour point	ASTM D97	°C	−39
Flash point, PMCC	ASTM D93	°C	197
Sulphated ash	ASTM D874	% wt	0.69

The UELR-5-1S linear electron accelerator was used for the preparation of the experiment. The mentioned accelerator ’s nominal energy of accelerated electrons is 5 MeV, and a maximum dose rate is 0.2 kGy/s. In order to detect the absorbed dose of ionizing radiation, we used a dosimetry system based on B3 radiochromic foils for which the manufacturer declares a measurement accuracy of 3% in the range of 3–100 kGy. If the value exceeds 100 kGy, it is possible to evaluate the dose with accuracy of 5%^13^.

**Figure 1:**
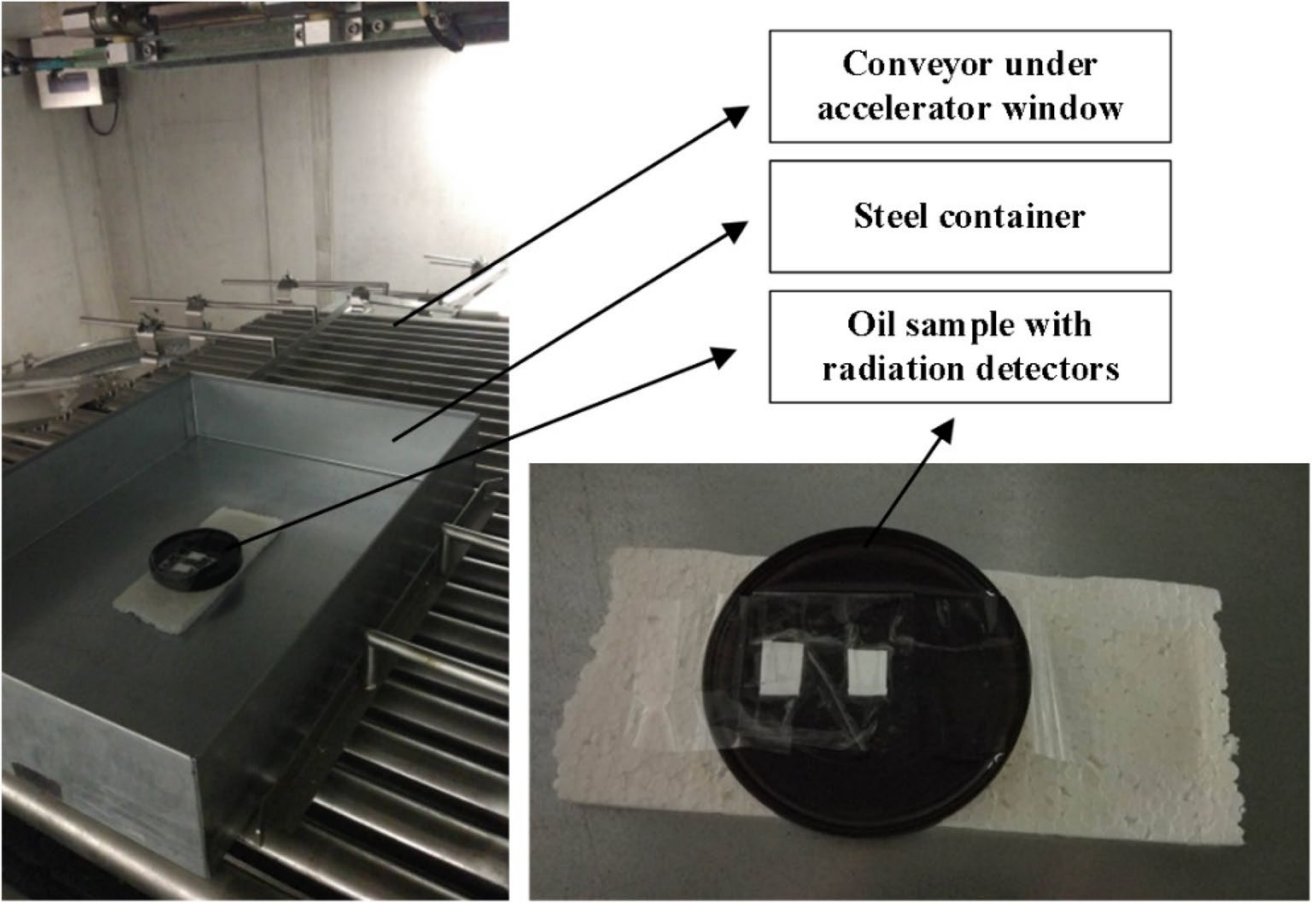
Oil sample prepared for irradiation process and a detail focusing on positioned dosimeters^14^.

Before irradiating the engine oil, which is part of the experiment in this article, it is necessary to determine a suitable irradiation container, depth profile, dose profile, and dose rate of electron radiation for the examined engine oil. Following the results of the four parameters mentioned above, it was possible to determine the optimal absorbed dose, irradiation parameters and geometry with respect to the maximum possible volume that can be irradiated in a certain container. Taking into account the results of previous experiments^14–17^, it is possible to determine the radiation dose of 28 kGy. Figure 1 shows the device that was used to irradiate individual samples.

Following the previous measurements, the radiation geometry is determined in a glass Petri dish of a 30 mm wall height and a 200 mm diameter into which 540 ml of engine oil is poured. The mentioned volume is reached at an 8 mm height which guarantees homogeneity of the absorbed dose in the oil volume in the range of up to 10%. The Petri dish is placed in a steel crate with a length of 550 mm, a width of 400 mm and a wall height of 100 mm, located on a conveyor belt 48 cm from the accelerator window. The accelerator parameters were set at accelerated electron energy of 5 MeV, a dose rate of 0.2 kGy/s, an electron cluster frequency of 240 Hz, a scanning frequency of 1 Hz with a width of 40 cm, a conveyor belt speed of 2.2 mm/s, and a beam current of 145 ± 3 μA. With the given settings, the samples were irradiated to a value of 23.7 kGy. The engine oil under examination was irradiated at a volume of 6 L, due to the necessary filling and topping of the engine during the exchange interval.

The engine oil capacity for the given internal combustion engine is 4.5 L. The rest of the irradiated oil was stored in the original motor oil container. The dates of the irradiation and measurement of the samples are shown in Table 2.

**Table 2:** Dates of irradiation and measurement of samples.

Monitored attribute/measuring method	Date of irradiation	Date of measurement
Kinematic viscosity, conv	–	20 April, 2021
Kinematic viscosity, e-beam	19 February, 2021	19 August, 2021
AES-RDE, conv	–	19 April, 2021
AES-RDE, e-beam	19 February, 2021	19 August, 2021
IR spectrometry, conv	–	19 April, 2021
IR spectrometry, e-beam	19 February, 2021	19 August, 2021
Particle analysis, LNF-C, conv	–	20 April, 2021
Particle analysis, LNF-C, e-beam	19 February, 2021	19 August, 2021

Engine oil samples were taken at intervals of 2000 km of the raid. The exact sampling intervals for conventional engine oil are shown in Table 3 and the sampling intervals for irradiated engine oil are shown in Table 4. The samples were taken after reaching the operating temperature of the coolant and driving at least 10 km more for the homogeneous heating of the engine oil.

**Table 3:** Dates of irradiation and measurement of samples.

Sample number	Date of sampling	Engine oil raid (km)	Car total raid (km)
Oil change	15 February, 2020	0	298,786
1	20 February, 2020	1029.5	299,816
2	05 March, 2020	2006.7	300,793
3	17 April, 2020	4052.3	302,838
4	13 May, 2020	6002.4	304,788
5	17 June, 2020	8028.0	306,815
6	10 August, 2020	9913.6	308,700
7	01 September, 2020	12,079.9	310,867
8	21 September, 2020	14,048.7	312,915
9	15 October, 2020	15,756.4	314,543

**Table 4:** Dates of irradiation and measurement of samples.

Sample number	Date of sampling	Engine oil raid (km)	Car total raid (km)
Oil change	20 February, 2021	0	320,960
1	24 February, 2021	1005.6	321,965
2	03 March, 2021	2598.4	324,563
3	16 March, 2021	5423.6	326,383
4	28 March 2021	7697.9	328,657
5	18 April, 2021	9961.1	330,921
6	07 May, 2021	12,083.1	333,043
7	28 May, 2021	14,101.6	335,061
8	18 June, 2021	16,058.8	337,018

The experiment was carried out for over two years, during the same seasons, from spring to fall. The beginning of monitoring of both conventional and irradiated engine oil was planned for the end of February which is almost the end of the winter period. The addition of the engine oil level after the low OBD was carried out twice during the technical life of both compared engine oils. The topping up was done during almost the same run-in. The date, mileage and amount of engine oil topping up are shown in Table 5. Driving conditions were maintained as much as possible for both monitored oils. It was driven in an urban environment and on the highway at approximately the same rate 50% each. Urban and highway driving was evenly distributed throughout the experiment. The vehicle used for the experiment is equipped with diesel engine, 1.9 L volume with power output 96 kW and P-D injection type.

**Table 5:** Dates of irradiation and measurement of samples.

Number/type of monitored oil	Date	Engine oil run-in (km)	Amount of engine oil (ml)
1/conventional	23 April, 2020	4740	650
2/conventional	10 August, 2020	9913	650
3/irradiated	18 March, 2021	5892	650
4/irradiated	14 May, 2021	12,725	650

During the entire experiment, the same filters of MANN-FILTER producer were used. The oil, air, fuel and cabin filters were changed before starting the experiment with both compared engine oils. We used filters from the given manufacturer which were compatible with the designated internal combustion engine. The filters used were oil filter HU 726/2X, fuel filter WK 853/3X, air filter C 27 192/1, and cabin filter CU 3037. No additives were applied to engine oil or fuel during the entire experiment, not even during the winter period when a new conventional engine oil of the same brand, type and kind was temporarily applied. Fuel was pumped mainly at Shell gas stations with the Shell FuelSave Diesel label.

The evaluation of kinematic viscosity was performed by the laboratory semiautomatic viscometer SpectroVISC Q300. Viscosity index is evaluated from kinematic viscosity by EN ISO 2909:2002 standard. Soot content, sulphation, oxidation, total base number and water content were measured by the portable fluid condition monitor FluidScan Q1000. The measurement of the content of chemical elements was carried out by the atomic emission spectrometer with rotation disc electrode Spectroil Q100. FTIR analysis was performed by the laboratory FTIR analyser apparatus Spectro-FTIR Bruker Alpha Q410. The total number of particles and statistical processing of the classified wear particles were evaluated by the automatic laser particle counter Spectro LNF-C Q200.

## Results

**Figure 2:**
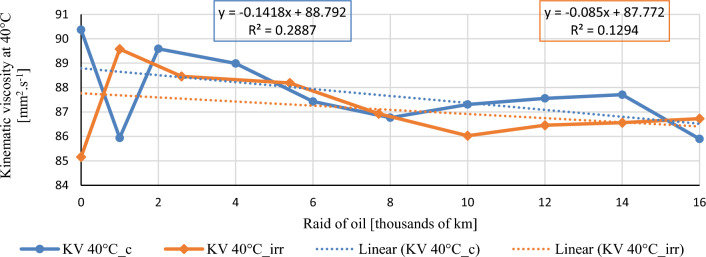
Graphical comparison of the kinematic viscosity of the reference and investigated oil at the temperature of 40 °C.

**Figure 3:**
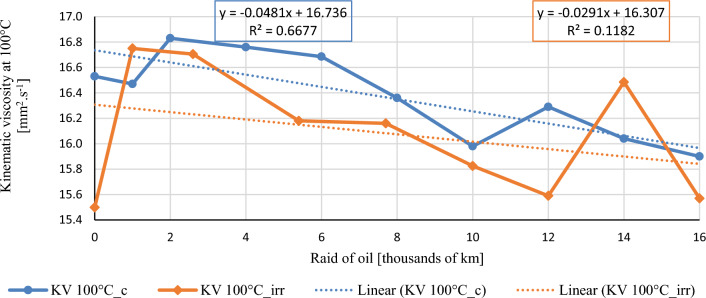
Graphical comparison of the kinematic viscosity of the reference and investigated oil at the temperature of 40 °C.

**Figure 4:**
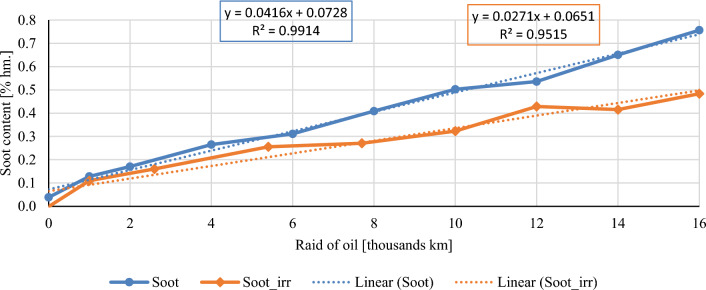
Graphical comparison of the viscosity index of the reference and investigated oil.

**Figure 5:**
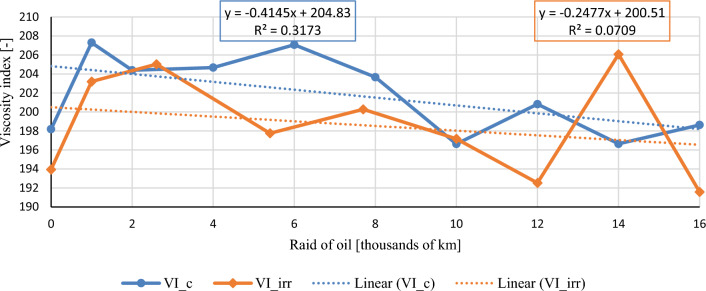
Graphical comparison of the soot content of the reference and investigated oil.

**Figure 6:**
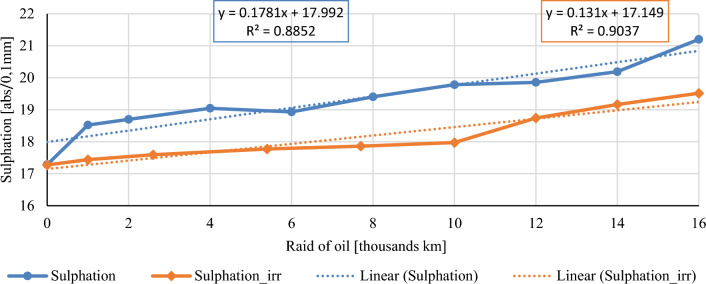
Graphical comparison of sulphation of the reference and investigated oil.

**Figure 7:**
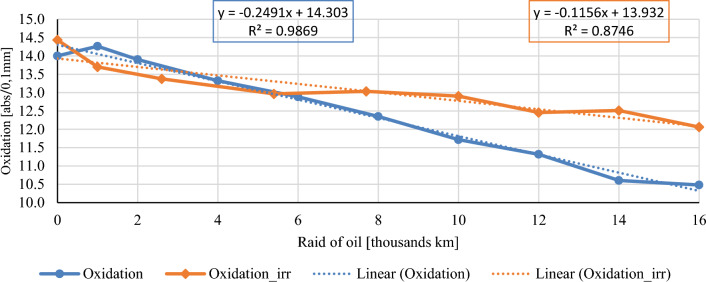
Graphical comparison of oxidation of the reference and investigated oil.

**Figure 8:**
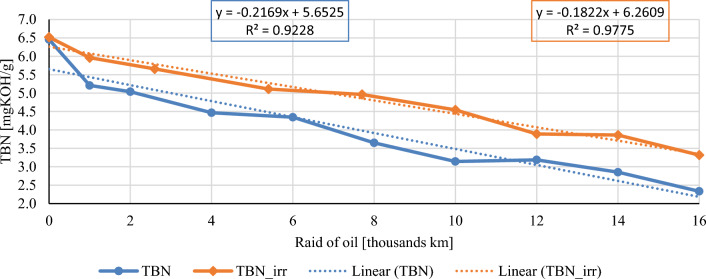
Graphical comparison of total base number of the reference and investigated oil.

**Figure 9:**
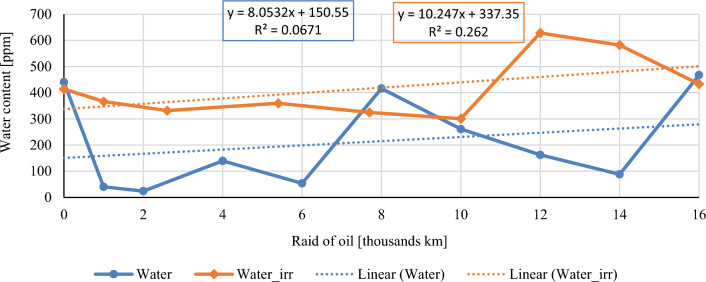
Graphical comparison of water content of the reference and investigated oil.

**Figure 10:**
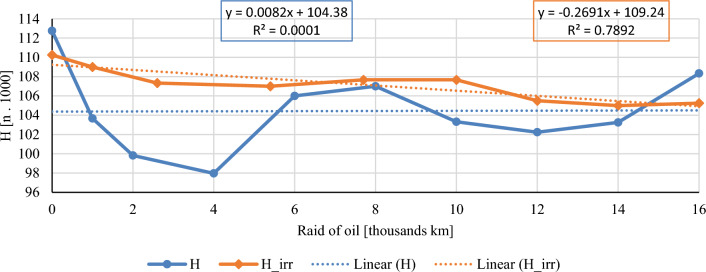
Graphical comparison of hydrogen content of the reference and investigated oil.

**Figure 11:**
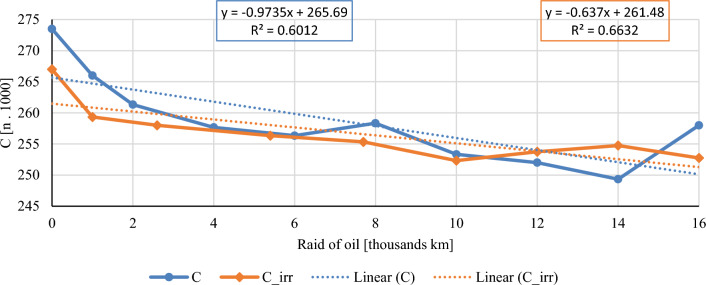
Graphical comparison of carbon content of the reference and investigated oil.

**Figure 12:**
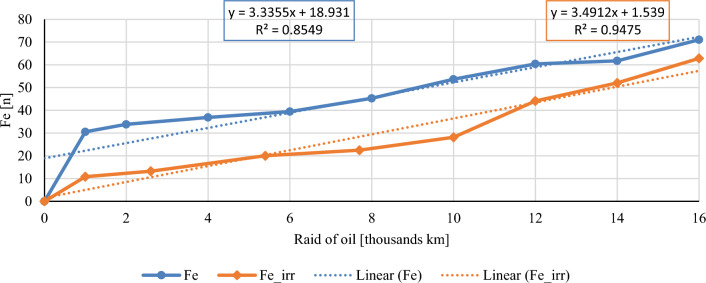
Graphical comparison of iron content of the reference and investigated oil.

**Figure 13:**
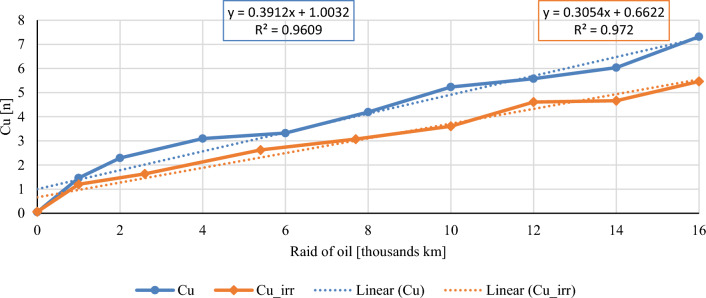
Graphical comparison of copper content of the reference and investigated oil.

**Figure 14:**
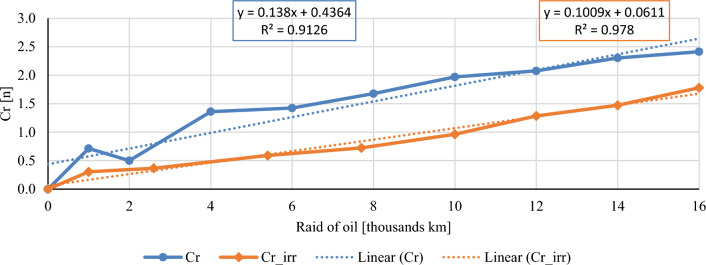
Graphical comparison of chrome content of the reference and investigated oil.

**Figure 15:**
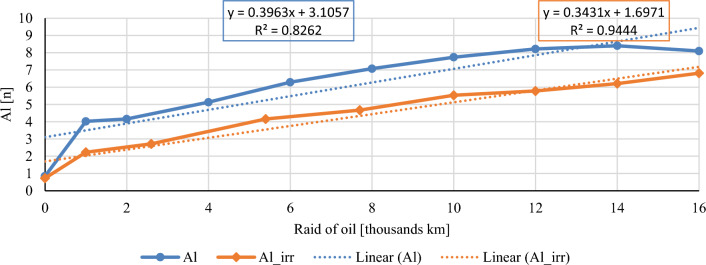
Graphical comparison of aluminium content of the reference and investigated oil.

**Figure 16:**
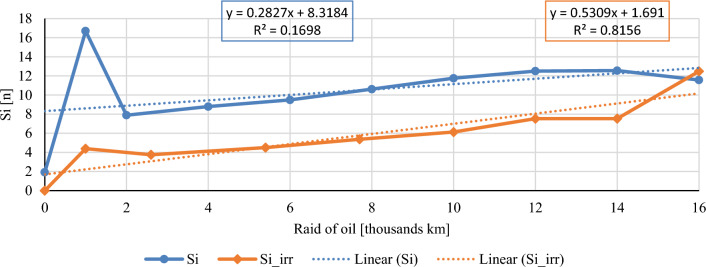
Graphical comparison of silicon content of the reference and investigated oil.

**Figure 17:**
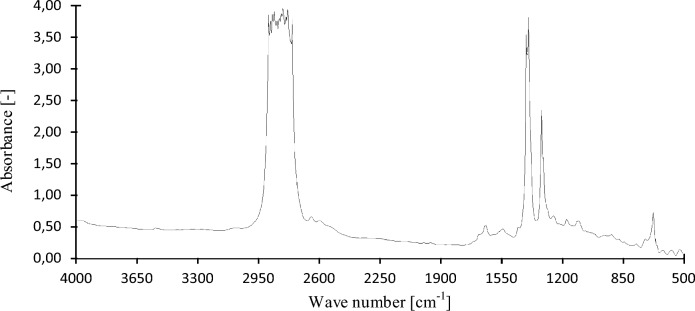
Graphic evaluation of FTIR analysis of the reference oil.

In this chapter, the measurements are made of the reference and investigated (irradiated) Castrol EDGE engine oil. Figure 2 shows a comparison of the kinematic viscosity at 40 °C, Fig. 3 shows the kinematic viscosity at 100 °C and compares the viscosity index over the lifetime of both engine oils. Furthermore, the measurements and comparisons were made of the soot content (Fig. 4), sulfation (Fig. 5), oxidation (Fig. 6), total TBN alkalinity (Fig. 7), water content (Fig. 8), hydrogen content (Fig. 9), carbon content (Fig. 10), iron content (Fig. 11), copper content (Fig. 12), chromium content (Fig. 13), aluminium content (Fig. 14), silicon content (Fig. 15), other impurities using FTIR analysis (Figs. 16 and 17). Finally, a comparison of the total number of particles in the reference and investigated engine oil is made.

It is desirable that the value of the kinematic viscosity at the temperature of 40 °C be as low as possible, thus closer to the value of the kinematic viscosity at the temperature of 100 °C. Figure 2 shows the course of the kinematic viscosity at 40 °C during the engine oil change intervals. The standard sampling error of the mean in the measurements did not exceed the value of 0.43 mm^2^/s. In the Figure, it is possible to see relatively large changes after the first 1000 km of both conventional and modified engine oil. As the following graph shows, the irradiated engine oil has a more stable kinematic viscosity for the next engine oil samples. This is also evidenced by the linear regression equation which has a parameter × closer to the zero value. During the first change of engine oil, a decrease in kinematic viscosity is observed, and, on the contrary, during the second change, a slight increase is observed.

Figure 3 shows the course of the kinematic viscosity of the engine oils measured at 100 °C during the exchange intervals. The standard sampling error of the mean in the measurements did not exceed the value of 0.17 mm^2^/s. The trend line shows a lower dependence of kinematic viscosity on raid, which is just as desirable as for kinematic viscosity at 40 °C. The viscosity value of the irradiated engine oil increases rapidly in the first 1000 km, which may be the reason for the mixing of the conventional residual engine oil left in the crankcase and in the turbocharger lubrication pipe during the engine oil change. During the first topping up of engine oil, a steady value of kinematic viscosity is observed, and during the second topping up, a rapid increase is observed for the irradiated engine oil. It should be noted that the values of kinematic viscosity at 100 °C during the raid for conventional engine oil are more stable than for irradiated oil, which is also proven by the linear regression line.

The differences in viscosity indexes are not significant, as can be seen in Fig. 4. The linear regression lines of both conventional and irradiated engine oil are almost identical, with a difference of 4 units of displacement of the line position. It follows that there is only a small possibility of weak intermolecular Van der Waals forces, which, among other features, also determine the resulting surface tension and viscosity of liquids. The recombination of hydrocarbon chains in combination with branching during irradiation is considered to be a more significant influence from this point of view. Emerging radicals, broken off shorter segments of chemical chains with an unpaired electron cannot exist independently; they react by pairing with electrons of another radical, resulting in a new chain which has a different arrangement, affecting the values of kinematic viscosity and viscosity index. During the first topping up of engine oil, slight increases in the viscosity index are observed. During the second topping oil level, we observed a rapid increase for the irradiated engine oil. The aforementioned recombination of hydrocarbon chains in connection with radical reactions is considered to be an important factor in the explanation of the above-mentioned phenomenon. The above data shows the need for a closer examination of the storage conditions of irradiated oils.

Soot is classified as mechanical impurities that are created in the combustion chamber of an internal combustion engine, especially during the combustion of diesel fuel. Soot is a product of incomplete combustion of diesel fuel. Most of the soot is pushed out of the combustion chamber with the exhaust gases. A small percentage of the soot enters the crankcase together with the exhaust gases. Soot and exhaust gases come into contact with engine oil in the crankcase and affect its properties. In the case of incorporating the exhaust gas recirculation system into the combustion engine concept, part of the exhaust gases is directed back into the intake pipe of the combustion engine, which increases the amount of soot in the combustion chamber. Engine oil is therefore more burdened by soot content. Figure 5 shows the trend of the soot content in conventional and irradiated engine oil. There is considerable variation between soot concentrations. Conventional engine oil is more stressed by its soot content by almost 0.3%. The increased soot content is manifested by the increased kinematic viscosity of the engine oil. The mentioned phenomenon is observed in Figs. 1 and 2, where the values of kinematic viscosity are mostly placed higher. The formation of soot is inversely proportional to the formation of nitrogen oxides NOX. For this reason, the alkalinity, oxidation, and nitrogen content in engine oil must also be assessed in connection with soot.

Sulphur in engine oils occurs only in small percentage of weight. It is contained in engine oils and fuels as an impurity during their production. Sulphur compounds and sulfur oxides or sulfates are acidic products that have a negative effect on the alkaline reserve, oxidation, and kinematic viscosity of engine oils. Figure 6 shows a graphical comparison of sulphation of conventional and irradiated engine oil. It is possible to see a clearly higher concentration of sulfates in conventional engine oil throughout the oil exchange interval, except at the beginning, where both monitored oils have the same value. The course of the sulphation products is almost linear in irradiated engine oil. The difference in the concentrations of sulfuration products increases with increasing mileage.

Figure 7 describes the comparison of the oxidation of conventional and irradiated engine oil. At the beginning of the experiment, the samples differ by negligible values. However, a noticeable decrease in conventional engine oil is evident in the oxidation values. The samples of irradiated oil show more stable values during the oil raid. As oxidation is supported by an increase in the temperature and viscosity index values for irradiated engine oil, increased resistance to oxidation could be expected. Oxidation is also affected by sulphation and nitration, that is, nitrogen oxides NOX and sulfur oxides SOX. In Fig. 6, a lower course of sulfate content in irradiated engine oil was described, which also predicts a lower stress on the oil by oxidation. The effect of topping the oil level with conventional engine oil does not affect the oxidation value. During the first and second topping up of the irradiated engine oil, the stabilization of the oxidation value is observed until the next sample collection.

The course of the total base number of conventional and irradiated engine oil is shown in Fig. 8. The total base number unites the substances contained in engine oil of a more or less alkaline nature. In a simplified way, it is possible to claim that a large amount of weakly alkaline products will express the same TBN value as a small amount of high-alkaline substances. This fact often makes it difficult to assess the impact of products that affect TBN, as it is not possible to determine exactly what substances are involved in the alkaline reserve of the investigated oil, therefore it is necessary to carry out associated experiments for further appropriate investigation. In Fig. 8, it is possible to observe a more significant decrease in the TBN value in the conventional engine oil compared to the irradiated.

According to the measured values, the beginning is almost identical, and the first 1000 km were decisive, where a striking difference can already be seen. Subsequently, throughout the course, the regression lines are almost parallel with a high coefficient of determination, which in both cases is above the level of 0.92 taking into account the sample of engine oil before pouring into the crankcase. With two oil level top-ups of both monitored oils, the stabilization of the TBN value is observed.

Figure 9 is an informative graph that shows the course of water content in the monitored engine oils. As the glycol content was measured at zero value (glycol values were not measurable), the measured water content comes only from air humidity and products of combustion. Based on the shape and position of the curve, it is clear that the combustion engine, during the experiment, worked mostly at operating temperatures. Sampling conditions were identical. From the above stated, it is possible to express that a larger amount of water accumulates in irradiated motor oil. Water content in engine oil also negatively affects oil oxidation. However, it does not have a significant effect on the measured limit values which corresponds to the measured oxidation values shown in Fig. 7.

The graphical course of the hydrogen content in the examined oils is shown in Fig. 10. The standard sampling error of the mean did not exceed the value of 2660 ppm in the measurements. Taking into account the nature of the measurement, it follows that even the water present in the engine oil was decomposed into individual elements. For this reason, it is possible to observe the similarity of the shape of the curve of hydrogen and water content in the examined samples throughout the entire duration of the experiment. This similarity applies to both conventional and irradiated engine oil.

Carbon is present in the base of engine oils and fuels, which are made up of hydrocarbons, and it is also one of the structural elements of steel. The amount of carbon also affects the combustion of diesel fuel, where soot is formed due to incomplete combustion. The amount of soot reaches approximately 0.8% in conventional and 0.5% in irradiated engine oil. The stated values do not have a significant impact on the total value of the carbon content. Paradoxically, with irradiated engine oil, the concentration of soot is lower, and in Fig. 11 we observe a slight increase in the amount of carbon when driving from 12,000 km onwards. The standard sampling error of the mean did not exceed 6,848 ppm in the measurements. It could be claimed then that the increase in carbon content is caused by measurements inaccuracies.

Iron is the main structural element of the metal parts of the internal combustion engine. Iron is usually found in bearings, piston pin, piston rings, working cylinder, it can also be found in the cam from the camshaft and tappets. Due to the lower value of the friction coefficient of similar irradiated engine oils^1^, it is possible to assume a lower content of wear particles during the technical life of the engine oil. The course of the iron content in engine oil is shown in Fig. 12. The standard sampling error of the mean in the measurements did not exceed the value of 3 ppm. It is possible to see relatively parallel curves of iron content throughout the experiment, except the beginning. In the phase of the first thousand kilometres, three times more iron content accumulated in conventional engine oil than in irradiated engine oil.

Copper belongs to the structural metals of combustion engines. Copper is usually found in rolling and plain bearings, bronze parts, valve tappets and piston pin bushings. The copper content during the experiment is shown in Fig. 13. The standard sampling error of the mean in the measurements did not exceed the value of 0.33 ppm. The copper content curves are relatively comparable, however, throughout the experiment, the irradiated engine oil has a lower copper content than the conventional engine oil.

Chromium also belongs to the structural metals of combustion engines. Chromium is usually found in parts where it is necessary to provide a hard surface layer, such as piston rings, piston pin, cylinders and liners of some internal combustion engines, etc. The chromium content during the experiment is shown in Fig. 14. The standard sampling error of the mean in the measurements did not exceed the value of 0.14 ppm. Chromium content trends are relatively comparable, but during the entire experiment, the irradiated engine oil has a lower chromium content than the conventional engine oil.

Aluminium alloys have their great justification in the construction of internal combustion engines. Aluminium alloys are often used in constructions where it is necessary to reduce the weight of the part, whenever possible and justified. Aluminium has an order of magnitude higher thermal expansion than construction steel and is also a very significant wear element. For this reason, sliding sleeves made of lead or tin composition, or bronze, pressed into the aluminium body are used in the moving parts. Pistons and heads of internal combustion engines are usually made of aluminium, some roller and plain bearings have admixtures of aluminium as well as certain types of bushings. The aluminium content during the experiment is shown in Fig. 15. The standard sampling error of the mean in the measurements did not exceed the value of 0.31 ppm. The trends of the aluminium content are comparable, but during the experiment the irradiated oil had a lower aluminium content than the conventional engine oil with a significant difference during the first thousand kilometres.

Silicon is a hard metalloid that dust particles are made of. Silicon in higher concentrations and compounds causes severe abrasive wear of friction pairs. Pollution in the form of dust occurs in the engine oil as a result of manufacturing imperfections of air and fuel filters, and penetrates into the engine oil through the leaks in the combustion chamber. However, silicon in the form of silicon oxides can also be found in the construction materials of working cylinders of an internal combustion engine, in camshaft bearings, and in some antifreeze liquids there is silicon used as an additive. The silicon content during the experiment is shown in Fig. 16. The standard sampling error of the mean in the measurements did not exceed the value of 0.42 ppm. During almost the entire experiment, the silicon content in the irradiated engine oil is approximately the half of reference oil.

The graphic evaluation of the FTIR analysis of conventional oil through the replacement interval is shown in Fig. 17. The beginning of the baseline can be seen around the absorbance value of 0.55, which indicates pollution of the engine oil, especially with soot. The presence of water in petroleum oils manifests itself in the region of 3150–3600 cm^−1^, where a small indistinct peak can be seen. This fact was indicated in Fig. 8. Taking into account the wave number 2976 cm^−1^ to 2838 cm^−1^, the first intense absorption band can be seen together with four other strong absorption bands with a peak at the wave numbers 1468 cm^−1^, 1454 cm^−1^, 1377 cm^−1^ and 722 cm^−1^ which indicate a high content of alkanes with different vibrational states. The peak at wave number 2964 cm^−1^ indicates the asymmetric ν stretching of the CH3 group of *n*-decane C_10_H_22_, the peak at wave number 2925 cm^−1^ indicates the asymmetric ν stretching of CH2 group of *n*-decane C_10_H_22_, and at wave number 2865 cm^−1^ it indicates the symmetric ν stretching of the functional group CH3 of *n*-decane C_10_H_22_. The peak at wave number 2850 cm^−1^ indicates the symmetric ν stretching of the CH2 groups. For the above-mentioned *n*-decane, it is possible to observe a peak at wave number 1468 cm^−1^, which indicates the σ symmetric deformation of CH2 group. The opposite is the absorption band at 1454 cm^−1^ indicating the σ asymmetric deformation of CH3 group, and the absorption band at 722 cm^−1^ indicates alternating fluctuation of CH3 groups.

In the region of 1680–1750 cm^−1^ it is possible to see the absorption band indicating the oil oxidation and the presence of aldehydes and carbonyls. The presence of aldehydes is commonly visible in the regions of 2900–2680 cm^−1^ and 1740–1660 cm^−1^. The absorption band in the region of 1605 cm^−1^ indicates the presence of carbonyls. Wave numbers 1605 cm^−1^ together with 1377 cm^−2^ determine the content of polymers with the functional group N=O, respectively O–N=O and other nitrogen compounds. A less pronounced absorbance peak at a wavelength of 1230 cm^−1^ detects the presence of detergent-dispersant additives. A significant depletion of detergent additives and a decrease in the concentration of zinc dialkyldithiophosphates ZDDP can also signal a decrease in TBN. The concentration of ZDDP as phosphorus additives occurs normally in the range of 0.1–2.5%, and in IR spectra it is manifested at wave numbers of 950–1050 cm^−1^. The absorption band at wave number 1159 cm^−1^ determines the amount of contained sulphates and sulphate detergents, and the almost negligible peaks at 815 cm^−1^ and 750 cm^−1^ indicate only a minimal fuel content in the engine oil.

**Figure 18:**
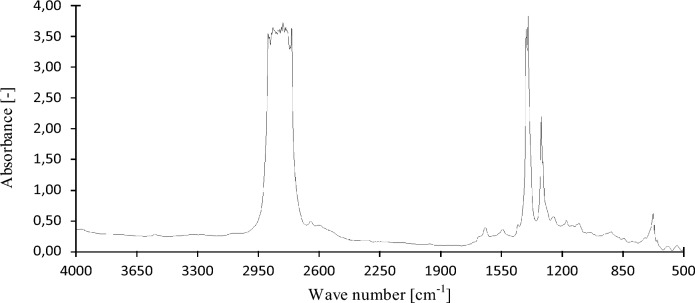
Graphic evaluation of FTIR analysis of the irradiated oil.

The graphic evaluation of the infrared analysis of the irradiated oil after the replacement interval is shown in Fig. 18. Based on the comparison of the initial course of the base line around the absorbance of 0.37 with the base line of the monitored conventional engine oil, it turns out that the entire course of the IR spectrum is lower. From the above, it is possible to express the lower total pollution of irradiated engine oil. The positions of the peaks of the individual absorption bands, which are placed below, are also related to the laying of the base line. The observable difference is in the comparison of the fuel content, which characterizes the absorption band in the region of 805–815 cm^−1^, where a lower absorbance value is observed. The three peaks of the absorption bands in the region of 700–550 cm^−1^, i.e., at the limit of the MIR spectroscopy measurability, have a lower absorbance value and are located towards lower values of the wave number.

**Figure 19:**
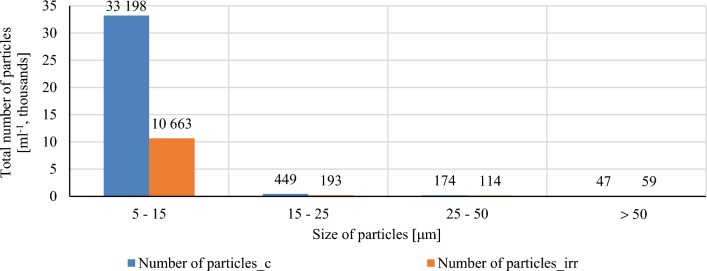
Graphical comparison of total number of particles of the reference and investigated oil.

A graphic comparison of the total number of wear particles with the distribution according to particle size is shown in Fig. 19. By comparing the number of wear particles, it is possible to express a relatively large difference in the monitored engine oils. In the case of conventional oil, a threefold higher number of particles in the size category 5–15 μm and an approximately twice higher number of particles with a size of 15–25 μm are detected. For larger particles, up to a measured size of 100 μm, the results are comparable. However, it should be noted that the used method of measuring with a laser particle counter has certain disadvantages. One of the most important fact is the issue of assigning particles without abrasive metal content, based on their shape, to individual categories according to the type of wear. The reason is often the almost zero light transmittance of the mentioned particles, which in the measuring cell gives a silhouette that the device's software considers a solid particle and classifies it in the appropriate category based on its shape. The stated disadvantage can be observed in the statistical processing of wear particles in Table 6.

**Table 6:** Statistical processing of wear particles with regard to the wear character and the material of the investigated particles.

Wear character/particles	Number of particles (ml^−1^)	Diameter (μm)	Standard deviation (μm)	The largest dimension (μm)
Abrasive	20.5	40.4	21.5	85.6
Abrasive e-beam	41.6	42.8	24.0	131.0
Wear	38.7	32.7	13.3	91.6
Wear e-beam	85.8	34.6	15.2	111.4
Fatigue	32.3	28.4	10.2	79.4
Fatigue e-beam	159.2	35.6	18.0	122.5
Non-metallic	85.0	36.4	20.5	133.8
Non-metallic e-beam	124.4	38.1	17.1	93.7
Unclassified	15.3	39.5	20.9	87.7
Unclassified e-beam	37.6	53.4	32.5	185.1
Fibres	16.0	–	–	–
Fibres e-beam	7.0	–	–	–

The number of particles and the basic statistics of wear particles with respect to the type of wear is shown in Table 6. In comparison, a greater number of wear particles of all categories are visible in the irradiated engine oil and they generally have a larger diameter. The number of particles of individual categories of conventional engine oil can be classified as a normal course of wear. The number of individual types of monitored wear particles does not exceed 40 particles in 1 ml. However, the number of fatigue particles in irradiated engine oil reaches 160 particles in 1 ml, which requires increased attention, as values above one hundred particles per 1 ml of the sample refer to the beginning of increased wear. However, the stated number of fatigue particles does not represent significant wear of the lubricating surfaces, but it is necessary to identify the reason for the measurement results.

## Discussion

The correlation coefficients R reached high values for almost all the observed samples, which also indicates using appropriate sampling method, appropriate methods of measurement and processing of results. With a new engine oil, it is necessary to take into account the impossibility of obtaining objective results due to the content of large molecules that wrap smaller functional groups, thus preventing the measurement of certain properties and elements. The above stated applies in particular to the measurement of detergent and dispersant additives.

Considering the measured values of the observed properties of conventional and irradiated engine oil, the effect of electron beam irradiation on their properties is obvious. The influence of radiation on the kinematic viscosity and thus also on the viscosity index is not as striking as it was assumed. However, it should be noted the possible effect of storage conditions on kinematic viscosity and viscosity index. Taking into account the measured values in the experiment, the positive influence of the second filling in the engine on the viscosity index is obvious.

As can be seen in Tables 3, 4, and 5, the irradiated engine oil level was topped up for the first time after one month from the start of the irradiated engine oil experiment and also after one month of irradiation. The second topping up was done three months after the start of the irradiated engine oil experiment. The above shows the need for a closer examination of the storage conditions of irradiated oils, as a longer storage period shows higher values of the viscosity index. Changes in the oxidation and total alkalinity number of the evaluated oils indicate a significant step in the experiment^18^. Oil oxidation and TBN belong to the basic evaluation elements used for determining engine oil change intervals. The measured values during the oil raid indicate a positive influence of electron radiation on the indicated values. With the results of kinematic viscosity, which show comparable values of chemical stability, it is possible to express the partial suitability of electron radiation for the modification of engine oils of internal combustion engines. Partly due to the conditional suitability of the modification and the complexity of engine oils, it is necessary to evaluate all aspects affecting the tribological system.

The course of the soot content shows a clear decrease in both the percentage share in irradiated engine oil and the measured sulphation values. The author evaluates the results of the mentioned two properties as a clearly positive effect of the e-beam modification. Taking into account the measured values of the friction coefficient for similar engine oils^1^, it is possible to assume lower wear in the used engine oil at the chosen dose of radiation, which is also confirmed by the measured amount of iron. Throughout the experiment, the iron content is lower than that of conventional engine oil, which is an important indicator for assessing the suitability of radiation treatment of engine oils. The first 1000 km oil raid are crucial, where the iron content of conventional engine oil has almost tripled. The following course of iron content is almost constant for both monitored engine oils. Copper content is another similar key indicator of the impact of wear. Copper is an important wear metal associated with wear, especially the wear of bearings and bushings which are heavily stressed in diesel combustion engines. An internal combustion engine with pump-nozzle injection has a higher load on the crankshaft and camshaft bearings due to the order of magnitude higher pressures in the working space compared to internal combustion engines using fuel injection by the help of a rotary pump. Despite the higher operating pressures of the combustion engine, the observed oil did not lose its lubricating properties during the radiation modification. The course of the content of copper as well as chromium and aluminium increases almost linearly from the beginning of the experiment, but the increase is more pronounced in the case of conventional engine oil.

The number of wear particles shown in Table 6 occurs as an unexpected measurement result. The number of particles from the abrasive, abrasion and fatigue wear mechanisms also show double concentration in 1 ml of the sample. A wear particle count above 100 is considered to be an increased wear and it is required higher attention while operating the engine again. With the values above 500 wear particles per 1 ml engine oil, the defect must be immediately searched and resolved. It follows from the above that the measured numbers of wear particles are not alarming, but it is necessary to identify the reason for the measurement results. Due to the nature of the experiment consisting in the basic research of the priority properties of the internal combustion engine oil and also as part of the feasibility study for subsequent ongoing research, the influence of the first investigated irradiated oil is possible. Since the internal combustion engine under investigation used to be lubricated with the same type of engine oil for a long time while not using any additives or flushing additives, we might consider a possible influence of deposits that were released when the modified engine oil was used, or to a certain extent they also dissolved into the oil filling. Taking into account the mentioned hypothesis, it is necessary to investigate more closely the chemical nature of irradiated engine oils and monitor the course of properties and content of irradiated engine oil during multiple use. The car is still drivable in the presence, without significant changes to the drive unit. The total number of particles shown in Fig. 19 is the number of all measured particles, not just wear particles. Taking into account the analysis of the wear particles and the fact of the usage of the same oil filters, there is no reason for the number of the smallest detectable particles to be significantly lower. The above implies a high probability of tearing, splitting and recombination of the hydrocarbon structure into shorter fragments. With regard to the measurement of kinematic viscosity, which is comparable to conventional engine oil, the probability of the formation of shorter chemical chains with longer side bonds, which, as in the case of detergent and dispersant additives, can be detected by the LNF-C measuring device and incorporated into the number statistics is possible particles. The mentioned hypothesis can be verified by measuring the number of particles of unused identical oils using a variation of the absorbed dose of radiation. Another method of verification can be the pumpability of the oil at low temperatures in combination with the determination of the flash point of modified engine oils.

The investigation of the influence of radicals is a complex process during which the attention must be paid to the amount and type of radicals. The reactivity of radicals is influenced by a number of factors that contribute to the formation of other radicals. The mentioned factors include the diversity of chemical elements and their concentrations, chemical bonds by which they are connected into molecules. The concentration of elements determines the probability with which they can further bind. A substantial part of the radical reactions takes place in the order of milliseconds to units of seconds during the irradiation. Based on the experiment, it is possible to express some conclusions by examining the influence of radicals on a theoretical level. It is generally accepted that the process of radical reactions is accompanied by three stages: initiation, propagation and termination^8^. In the initiation stage, two cases can occur: ionization and excitation of atoms and molecules. In the case of ionization, the polymer is ionized and immediately after the interaction, the ionized molecule dissociates into a radical. During excitation, the irradiated atom passes from an equilibrium state to an excited state. When returning to the equilibrium state, the electron emits energy in the form of X-rays. Alternatively, the second possibility occurs in which the excited molecule dissociates into a radical. The mentioned chemical reactions complete the initiation stage and the second phase—propagation begins. The two radicals formed at the initiation stage return to a stable state by binding to another molecule by removing an electron, which creates another radical. Radicals formed on primary carbon are more reactive than radicals on secondary carbon. The terminal stage occurs in the case of a reaction between two radicals. In this manner, the radical returns to a stable state by binding to another suitable radical in sufficient proximity. By the mentioned mechanism, the reaction cycle of the propagation stage is interrupted and the chain reaction is terminated. Terminal steps rarely occur, as the concentration of radicals at any given moment is very low.

Related to the amount of contained hydrocarbons and additives added to the engine oil, a large number of radical reaction products are formed. Following the comparison of the IR spectra shown in Figs. 17 and 18, it is possible to see the differences in the measured absorbance values of individual hydrocarbon derivatives. In the case of irradiated engine oil, the number of n–decane is higher in the area 3000–2800 cm^−1^, 1460 cm^−1^ and 720 cm^−1^ compared to the baseline. Alkanes are used in engine oils in conjunction with base oils. The content of alkanes forms a significant part of the fundamental property of oils—lubrication. The quantity and quality of alkanes together with additives used for optimizing lubricity properties are directly related to the friction coefficient of the tribological system of which they are part. The absorbance band in the region of 1377 cm^−1^ indicates the compounds of alkanes with nitrogen. However, nitration of engine oil can also occur during the natural run-in of engine oil by replacing the hydrogen atom with the nitro group by NO2, which is most often manifested on primary carbons. Nitrogen oxides NO2 are created by incomplete combustion of fuel in the combustion chamber of an internal combustion engine. The increased content of aldehydes can be observed by absorption bands at wave numbers of 2727 cm^−1^ and 1706 cm^−1^. The physical properties of aldehydes directly depend on the molar mass. The higher number of carbons in aldehydes is directly dependent on the boiling point. The reactivity of aldehydes is relatively high but decreases with increasing length of the chemical chain. The reactivity of aldehydes is caused by the presence of a carbonyl group which causes a higher electronegativity of oxygen in the functional group. It should be noted that in addition to the mentioned reaction mechanism of radical reactions in the process of irradiation with high-energy electrons, the occurrence of pericyclic reactions is also possible, affecting the entire process of modifying the properties of engine oils.

## Conclusion

The influence of electron radiation on engine oil was described by the selected monitored properties during the engine oil change interval. Considering the extent of the work, there were more monitored properties, however, only the most significant ones have been listed in the article. A significant step has been achieved in the research of viscosity index, oxidation, TBN and the number of particles of internal combustion engine wear.

Evaluating the optimization quality of radiation-modified engine oil is difficult, as there are currently no values for the appropriate absorbed dose of electron radiation for the monitored properties and the chosen type of oil. In light of these facts, it will be necessary to perform further numerous experiments and measurements of engine oils properties and evaluate the obtained information by comparative methods with conventional engine oils. In order to create an effective procedure with the application of ionizing radiation in the development of engine oils, it is necessary to meet a number of prerequisites, which include: a source of ionizing radiation with high accuracy of transverse and longitudinal homogeneity of the beam, creation of a suitable conveyor system for liquids with regard to ensuring uniform flow during irradiation at each point of the cross-section of the flow, a reliable and accurate dosimetric system, operation of a number of test rooms and tested combustion engines in laboratory conditions as well as vehicles in real operation for thorough statistical evaluation, regular collection of the examined oils samples at specified intervals, measurement of all examined oils immediately after taking the sample, creation of data database for various combustion engines and engine oils, examination of engine oils from the point of view of their origin (type of base oil) and additive package, evaluation of different storage conditions and their effect on the properties of engine oils. Subsequently, it will be possible to create models of the suitability of modified oils for different types of combustion engines which are significant in terms of the absorbed doses of radiation and added additives in base oils. Based on the results of the research, it could be concluded that the selected method is suitable for modifying the properties of lubricating oils of internal combustion engines.

## Data Availability

All data generated or analyzed during this study are included in this published article.
